# Challenging frontiers in neuroplastic cranial reconstruction: addressing neurosurgical wound healing complications through interdisciplinary collaboration – an observational study

**DOI:** 10.1007/s00701-024-06328-z

**Published:** 2024-10-29

**Authors:** Kathrin M. Aufschnaiter-Hiessboeck, Harald Stefanits, Tobias Rossmann, Martin Aichholzer, Wolfgang Senker, Philip Rauch, Helga Wagner, Philipp Hermann, Matthias Gmeiner, Andreas Gruber, Manfred Schmidt

**Affiliations:** 1 Department of Neurosurgery, Kepler University Hospital and Johannes Kepler University Linz, Wagner-Jauregg Weg 15, 4020 Linz and Altenbergerstrasse 69, Linz, 4040 Austria; 2 Center for Clinical Studies (CCS Linz), Johannes Kepler University Linz, Krankenhausstraße 5, 4020 Linz and Altenberger Strasse 69, Linz, 4040 Austria; 3 Plastic and Reconstructive Surgery, Kepler University Hospital, Krankenhausstrasse 9, Linz, 4020 Austria; 4 Department of Applied Statistics, Medical Statistics and Biometry, Johannes Kepler University, Altenberger Strasse 69, Linz, Linz, 4040 Austria; 5 Clinical Research Institute für Neurosciences, Faculty of Medicine, Johannes Kepler University Linz, Wagner-Jauregg Weg 15, Linz, 4020 Austria

**Keywords:** Free flap, Necrosis, Question mark shaped incision, Scalp reconstruction, Wound dehiscence

## Abstract

**Background and objectives:**

Although rare, complications like skin dehiscence and necrosis after neurosurgery pose significant challenges by increasing the risk of infections spreading to the epidural, subdural, or intracerebral spaces. This retrospective, single-center study aims to assess the prior clinical courses, neuroplastic repair, and outcomes of  patients with skin defects following cranial neurosurgical procedures, and to outline our interdisciplinary reconstructive protocol.

**Methods:**

A retrospective analysis was performed on cranial surgeries conducted at the Department of Neurosurgery,  spanning from 2017 to 2023. Patients with skin defects requiring the combined expertise of neurosurgery and plastic surgery for effective treatment were included. The sizes of the skin defects were measured using intraoperative photographs analyzed with the freeware ImageJ software, version 2018. All patients provided informed consent for the surgeries. If informed consent was not possible due to neurological deterioration, consent was sought from adult representatives or next of kin except for acute circumstances. All patients admitted to our hospital agree to the pseudonymized use of their medical data and tissue specimens for research purposes in their treatment contract.

**Results:**

A cohort of 24 patients experiencing wound healing complications after neurosurgical procedures underwent a total of 29 interdisciplinary surgeries for the reconstruction of skin, dural, and bone defects.  After the neuroplastic surgery, 8 out of 24 patients (33.3%) developed surgical complications, with 6 of these requiring revision surgeries due to persistent cranial infection.  In all cases,  permanent wound closure was successfully achieved following adherence to the proposed treatment algorithm.

**Conclusions:**

Our study underscores the necessity of an integrated neurosurgical and plastic surgical approach to effectively manage wound healing complications in a single stage surgery. Key interventions include differentiation between necrosis and gaping lesions, alongside precise management of neurosurgical issues like cerebrospinal fluid fistulas and hydrocephalus. Plastic surgical expertise in assessing the possibilities and limitations of both local and free flap surgeries is essential.

## Introduction

Causes of cranial wound healing disorders and regimens for scalp defect reconstructions have been described in the literature [5, 6, 9, 15, 17–19, 23, 24, 26]. Predisposing factors and comorbidities, including cerebrospinal fluid (CSF) leak, diabetes mellitus, and prior irradiation, have been recognized as fundamental contributors to the development of postoperative wound healing disorders [4, 15, 18, 23, 28]. Additionally, question mark shaped incisions [16, 27] (widely used for decompressive craniectomies) as well as extracranial-intracranial bypass surgeries [4, 24], are known contributors to skin necrosis due to the partial disruption of the arterial supply to the skin flap.

In patients with a history of multiple surgeries, challenges such as complex scar formations, diminished skin elasticity, and wound margin inversion, complicate primary wound closure in subsequent revisions. These circumstances require plastic surgical techniques, including local or free flaps, chosen based on the quality of surrounding skin and the size of the defect. Additionally, infections in deeper cranial areas, potentially involving open paranasal sinuses or mastoid air cells, along with dural defects, demand neurosurgical intervention. An integrative, interdisciplinary approach can thus enable addressing these complexities in a single surgical session, enhancing patient outcomes [1, 11, 15, 20]. Leveraging our interdisciplinary expertise with this heterogeneous patient cohort, our goal was to formulate a treatment algorithm.

## Methods

We retrospectively analyzed patients operated between January 1, 2017, and December 31, 2023, who presented with wound dehiscence or necrotic scalp lesions. All patients were treated through a collaborative effort between plastic and neurosurgery teams. In contrast to the relatively straightforward cranial reconstructions performed for skin tumors, our study addresses the significantly more complex scenarios involving patients with complications arising from neurosurgical procedures. This distinction is crucial as the presence of underlying neurosurgical issues presents unique challenges and necessitates a nuanced, interdisciplinary approach to achieve optimal outcomes [2]. Ischemic necrosis was defined as a grey/black discolorated and demarking skin area. Wound dehiscence primarily presented as circular or oval “hole like” openings, revealing underlying structures such as bone flaps, allografts, or the dura mater. Patients presenting with skin defects exceeding 1.5 cm², or exhibiting irradiated, thickened, thinned, and inelastic surrounding skin characteristics, underwent preliminary assessment by a plastic surgeon to evaluate the feasibility of achieving primary tension-free wound closure. In case primary closure appeared unfeasible, and additional plastic surgical expertise was needed, an interdisciplinary surgical strategy was formulated. This involved planning the ideal timing and extent of surgery, depending on the progression of necrosis demarcation and the presence of a CSF leak or further intracranial pathologies, as well as selection of the flap type and shape of skin incision. Surgeries were conducted interdisciplinarily from skin incision to skin closure.

### Radiology

Preoperative MR-scans were performed to evaluate intracranial abscesses, as these findings determined the urgency for surgery. Postoperative CT- and MR- scans were conducted to evaluate blood collections and follow intracranial infectious changes. In preparation for free-flap surgery, we included angio CT scans or Doppler ultrasound in the preoperative diagnostic evaluation to visualize the donor and recipient vessels.

### Surgery

At the request of the plastic surgeon, large skin areas were shaved to provide adequate space for skin mobilization and the potential for additional incisions. Skin shaving was performed using electric shavers, as disposable razors were avoided due to their tendency to damage superficial skin layers. Following the marking of the cranial skin incision, plastic and neurosurgical procedures were initiated concurrently, particularly in cases requiring the harvest of fascia lata or free flap preparation. Particular care was taken to avoid scratching the skin with the forceps, and skin wound clips were avoided due to their potential to compromise skin perfusion. Intraoperative bacterial swabs were taken. In most patients, careful debridement, including the removal of the bone flap or allograft, was performed. This was followed by smoothing of sharp bone edges using a high-speed drill to protect the overlying skin. Special care was taken to ensure closure of the paranasal sinuses and to achieve a watertight dural closure. Whenever possible, skin defects were covered with local flaps, whereas free flaps were reserved for large defects or when the surrounding skin quality was poor. A tension-free skin closure was paramount to ensure optimal blood supply to the wound margins. Clinical and radiological data were extracted from electronic medical records.

### Perioperative antibiotic regimen

In cases involving cerebrospinal fluid (CSF) leakage or extensive necrotic tissue, immediate antibiotic therapy was initiated. Standard perioperative broad-spectrum antibiotics were administered in all other cases. Microbiological swabs were collected intraoperatively, and following the availability of culture results and antibiogram data, targeted intravenous antibiotic therapy was administered for a minimum of one week before transitioning to oral therapy. Antibiotics were discontinued upon favorable progression of wound healing. In cases of persistent infection, antibiotic treatment was extended until clinical signs, laboratory values, and MRI findings confirmed the resolution of the infection.

### Skin defect size analysis

Sizes of skin defects were calculated from intraoperative photographs with the freeware ImageJ software version 2018 [21]. A ruler was either placed next to the skin defect intraoperatively, or the bony defect measured on preoperative CCT scans was used as a reference.

#### Statistical analysis

The primary outcome of interest was patient survival. Patient demographics (sex and age), underlying neurosurgical pathologies, characteristics of the skin defect (size and type, distinguishing between necrosis and non-necrotic defects), comorbidities, the time elapsed between neurosurgery and interdisciplinary surgical intervention, and the presence of a question mark-shaped incision were among the variables assessed. Nominal variables were summarized using absolute and relative frequencies. The distribution of continuous variables was described by the mean ± standard deviation and the range (minimum-maximum). Statistical analysis was performed using the statistical software R (Version 4.3.1).

## Results

Between January 1, 2017, and December 31, 2023, our institution performed 8,094 cranial surgeries, including burr holes for chronic subdural hematomas, shunt procedures, and more complex surgeries for tumor resections, trauma, or decompressive craniectomies. During the given period, 225 surgeries were performed due to wound healing complications, affecting 175 patients. Whilst most wound margins could be easily adjusted without the requiring interdisciplinary managemenent, 24 patients underwent a total of 29 surgeries involving both plastic surgery and neurosurgical teams. Patient characteristics are summarized in Table 1. 24 patients − 16 males (66.7%) and 8 females (33.3%) - aged 27.3 to 86.5 years (57.9 ± 15.3) required 29 interdisciplinary surgeries due to wound healing disorders with skin defects. Underlying neurosurgical pathologies were traumatic brain injury (TBI) in 13 patients (54.2%), intracranial tumor in 6 patients (25%), neurovascular diseases in 4 patients (16.7%), and hydrocephalus with prior ventriculo-peritoneal shunt implantation in 1 patient (4.2%). During the initial combined plastic and neurosurgical intervention, 7 patients (29.2%) were neurologically intact, another 6 patients (25%) were comatose, 5 patients (20.8%) were hemiparetic and 6 patients (25%) presented with other focal neurological deficits.

**Table 1:** Patient and surgery characteristics

Patient ID	Surgery ID	side	sex	age at interdisciplinary surgery	initial pathology	trauma flap (question mark incision)	ischemic necrosis	skin defect size (cm^2^)	flap type	defect site	survival time (days)
1	1	left	m	57	trauma	yes		11	occipital rotation flap	temporooccipital	dead after 150
1	2	right	m	-	trauma	yes		6	bipedicled flap	temporooccipital	-
2	3	left	m	50	cerebrovascular - EC/IC bypass	no		39.9	temporal rotation flap	frontotemporal	1328
3	4	left	m	67	trauma	yes		6.5	occipital rotation flap	parietooccipital	dead after 26
4	5	left	m	31	AVM - resection	yes		n/a	occipital rotation flap	parietooccipital	1176
4	6	left	m	-	heterologous CP	yes		no defect	flap reopening for CP	no defect	-
5	7	right	f	48	trauma	yes		38.1	occipital rotation flap	parietooccipital	925
5	8	right	f	-	autologous CP	yes		no defect	flap reopening for CP	no defect	-
6	9	right	f	43	intracranial tumor	no		0.8	temporal rotation flap	parietooccipital	813
7	10	right	m	74	trauma	yes		19.2	occipital rotation flap	parietooccipital	dead after 52
8	11	left	f	27	hydrocephalus - vp shunt	no		0.4	occipital rotation flap	temporal	1659
9	12	right	m	39	trauma	yes		11.1	skin expansion through galeal scoring and advancement flaps	parietooccipital	778
10	13	right	f	56	ischemic stroke	yes		3.5	skin expansion through galeal scoring and advancement flap	frontal	951
11	14	right	m	64	intracranial tumor	no		10.2	occipital rotation flap	temporal	757
12	15	left	f	47	intracranial tumor	no		1.8	skin expansion through galeal scoring and advancement flap	occipital	1435
13	16	right	m	60	trauma	no		3.4	skin expansion through galeal scoring and advancement flap	frontal	640
14	17	right	m	49	trauma	yes		2.7	skin expansion through galeal scoring and advancement flap	temporal	504
15	18	right	m	59	trauma	yes		7.6	skin expansion through galeal scoring and advancement flap	frontoparietal	473
16	19	right	m	67	intracranial tumor	no		29.8	free latissimus dorsi flap	frontotemporal	459
17	20	left	m	76	intracranial tumor	no		5.1	occipital rotation flap	frontal	441
18	21	left	m	85	trauma	yes		26.4	temporal rotation flap	frontotemporal	1693
19	22	right	f	49	intracranial tumor	no		n/a	SCIP-flap	temporal	dead after 266
19	23	right	f	-	intracranial tumor	no		5.9	skin expansion through galeal scoring and advancement flap	frontotemporal	-
19	24	right	f	-	intracranial tumor	no		no defect	direct closure	frontotemporal	-
20	25	left	m	86	trauma	yes		9.7	skin expansion through galeal scoring and advancement flap	parietotemporal	dead after 8
21	26	left	f	57	intracranial tumor	no		6.5	skin expansion through galeal scoring and advancement flap	frontal	61
22	27	left	f	70	subarachnoidal hemorrhage	yes		13.4	skin expansion through galeal scoring and advancement flap	frontotemporoparietal	22
23	28	left	m	65	trauma	yes		18.3	frontal transposition flap	frontoparietal	105
24	29	left	m	65	trauma	no		11.3	skin expansion through galeal scoring and advancement flap	frontal	36

*Abbreviations*: *AVM* arteriovenous malformation, *CP* cranioplasty, *EC/IC bypass* extracranial to intracranial bypass, *n/a* not available, *SCIP-flap* superficial circumflex iliac artery perforator flap, *vp shunt* ventriculoperitoneal shunt, survival time time between interdisciplinary surgery and of telefone interview or death,  „ - “  = same patient as line above

Cardiovascular comorbidities were present in 8 patients (33.3%), followed by depression in 6 patients (25%), leading to suicide attempts with traumatic brain injury in 2 patients (8.3%). A history of extraaxial malignant tumor was noted in 3 patients (12.5%), diabetes mellitus in 2 patients (8.3%), smoking, chronic lung disease and drug abuse were each detectable in 2 patients (8.3%), whereas none of the patients was adipose. 8 patients (34.8%) did not suffer from any apparent comorbidities. For one patient comorbidities were not documented.

### Pre-neuroplastic complications resulting in necrosis or wound gaping

17 of 21 patients (81%) who developed necrosis or wound gaping had at least one intra- and postoperative complication during or after the initial neurosurgical procedure. 5 patients (23.8%) had bleeding or rebleeding complications, in 5 patients (23.8%) a CSF-leak occurred after the previous neurosurgical treatment and 6 patients (28.6%) developed a mengingitis or ventriculitis. During their clinical progression, 7 patients (33.3%) experienced the development of a hydrocephalus. Three patients were previously operated in external institutions and therefore no exact data concerning complications was available.

The number of neurosurgical operations prior to interdisciplinary intervention ranged from 1 to 8 and was assessed in all 24 patients. Sixteen patients (66.7%) had undergone 2 or more neurosurgical procedures, and 5 patients (20.8%) had received prior tumor irradiation. Traumaflaps with a question mark shaped incision were found in 12 of the 24 patients (50.0%). In half of the patients, abscesses were identified via preoperative MRI scans or during surgery.

*Skin defect type* Skin defects appeared as necrosis in 11 of 24 patients (45.8%) and as wound dehiscence in 13 of 24 patients (54.2%). Notably, of the 11 patients with necrosis, 8 patients (72.7%) had a question mark shaped wound. Time intervals between the initial neurosurgical procedure and interdisciplinary surgery were shorter in the necrosis group than in the wound dehiscence group (9–95 days, 51.0 ± 35.7 vs. 13-7319 days, 1649.5 ± 2268.4).

*Skin defect sizes* ranged from 0.4 cm² to 39.9 cm² (12.6 ± 11.6 cm²). In two patients, no intraoperative photographs were taken, and therefore no defect size could be evaluated.

*Surgery* The average surgery duration for all patients was 171.30 min (51–424 min, 171.3 ± 99.2). The average intraoperative blood loss for all patients was 158 ml (100–500 ml, 158 ± 126).

In 24 out of 29 (82.8%) interdisciplinary surgeries, skin defects were covered with local skin flaps: 8 occipital rotation flaps (27.6%), 3 temporal rotation flaps (10.3%), 1 frontal transposition flap (3.5%), 11 skin expansions through galeal scoring and advancement flaps (37.9%) and 1 bipedicled flap (3.5%) (see Table 1). 2 skin defects (6.9%) were covered with free flaps: one superficial circumflex iliac artery perforator (SCIP) flap (defect size could not be determined) and one free latissimus dorsi muscle flap for a 30 cm [6] scalp defect. Both patients had a history of multiple previous intracranial tumor surgeries, radiation therapy and surgeries for wound infections (pat. 16 and pat. 19). In two trauma patients (8.3%), open paranasal sinuses or mastoid air cells were detected on preoperative CT scans and sealed intraoperatively. Fascia lata was harvested in 3 (10.3%) surgeries in order to close dural defects. In 3 cases (10.3%), the skin flap was reopened during an interdisciplinary follow-up surgery. In two cases (patients 4 and 5), bone defects were covered, while in one case (patient 19), an infected bone flap was removed, along with the resection of a recurrent intracranial tumor. For the first surgery of each patient, in 11 out of 24 patients (45.8%) the bone flap had already been removed in a previous surgery, in further 12 out of 24 patients (50%) the bone flap was removed in the neuroplastic procedure. Notably, only in the cases of patients 4 and 5, subsequent surgeries involved the implantation of one autologous and one heterologous bone flap, respectively. This decision was based on the patients’ younger age (32 and 48 years) and favorable neurological recovery.

### Germs and antibiotic regimen

In both the necrosis and skin defect patient groups, a wide spectrum of 19 different bacteria was identified, with 13 of these (68.4%) identified as typical skin flora. This included Staphylococcus aureus, Staphylococcus lugdunensis, Staphylococcus epidermidis, and Staphylococcus hominis. Notably, Methicillin-Resistant Staphylococcus epidermidis (MRSE) was exclusively detected in the necrosis group, affecting a significant 27.3% of patients (3 out of 11). The most frequently used antibiotics were cephalosporins, specifically Cefuroxim, Cefazolin, Cefepim, and Ceftriaxon. Additionally, Fosfomycin, from the phosphonic acid group, and Clindamycin, from the lincosamide group, as well as Meropenem, a broad-spectrum beta-lactam antibiotic, were also utilized.

### Hospital and ICU stay durations

The average hospital stay for our patients was 51.73 days (2–266 days, 51.7 ± 67.3) and the average ICU stay was 16.08 days (0–153 days, 16.1 ± 39.6). Patients with gaping lesions experienced considerably shorter hospital stays, averaging 17.93 days (2–55 days, 17.9 ± 14.6) and ICU stays with an average of 0.53 days (0–2 days, 0.5 ± 0.6). In contrast, those with necrosis had substantially longer hospital stays, averaging 97.82 days (2–266 days, 97.8 ± 83.5) and ICU stays averaging 37.27 days (0–153 days, 37.3 ± 55.4).

### Outcomes and complications

No early flap related complications like venous thrombosis or arterial insuffiency resulting in flap necrosis were recorded. Nevertheless, 8 out of 24 patients (33.3%) experienced other surgical complications: persisting cranial infection necessitated revision surgeries in 6 of these patients. In 3 patients, the infection was related to allografts: one patient had a ventriculoperitoneal shunt (pat. 11) and one patient had a bone flap (pat. 19) left in place during the first interdisciplinary surgery. One patient, with a previous history of bone flap explantation due to wound dehiscence and an epidural abscess, proceeded against our recommendation to delay covering the bone defect for at least 9 months: the bone defect was covered at an external institution only 4 months later. This premature intervention resulted in a new wound healing disorder and a septic condition within three months (pat. 14). One instance of epidural rebleeding occurred subsequent to galeal scoring and advancement flap. Revision surgery was performed without new neurological deficits (pat. 27). Another patient with a psychiatric history (pat. 28) exhibited destructive behavior towards the split skin graft, leading to visible periosteum. Through conservative treatment measures, successful healing was attained.

Ongoing infection, severe traumatic brain injury (TBI), and underlying malignancy resulted in a substantial mortality rate of 20.8% (5 out of 24 patients) within nine months following the reconstruction surgeries.

**Fig. 1:**
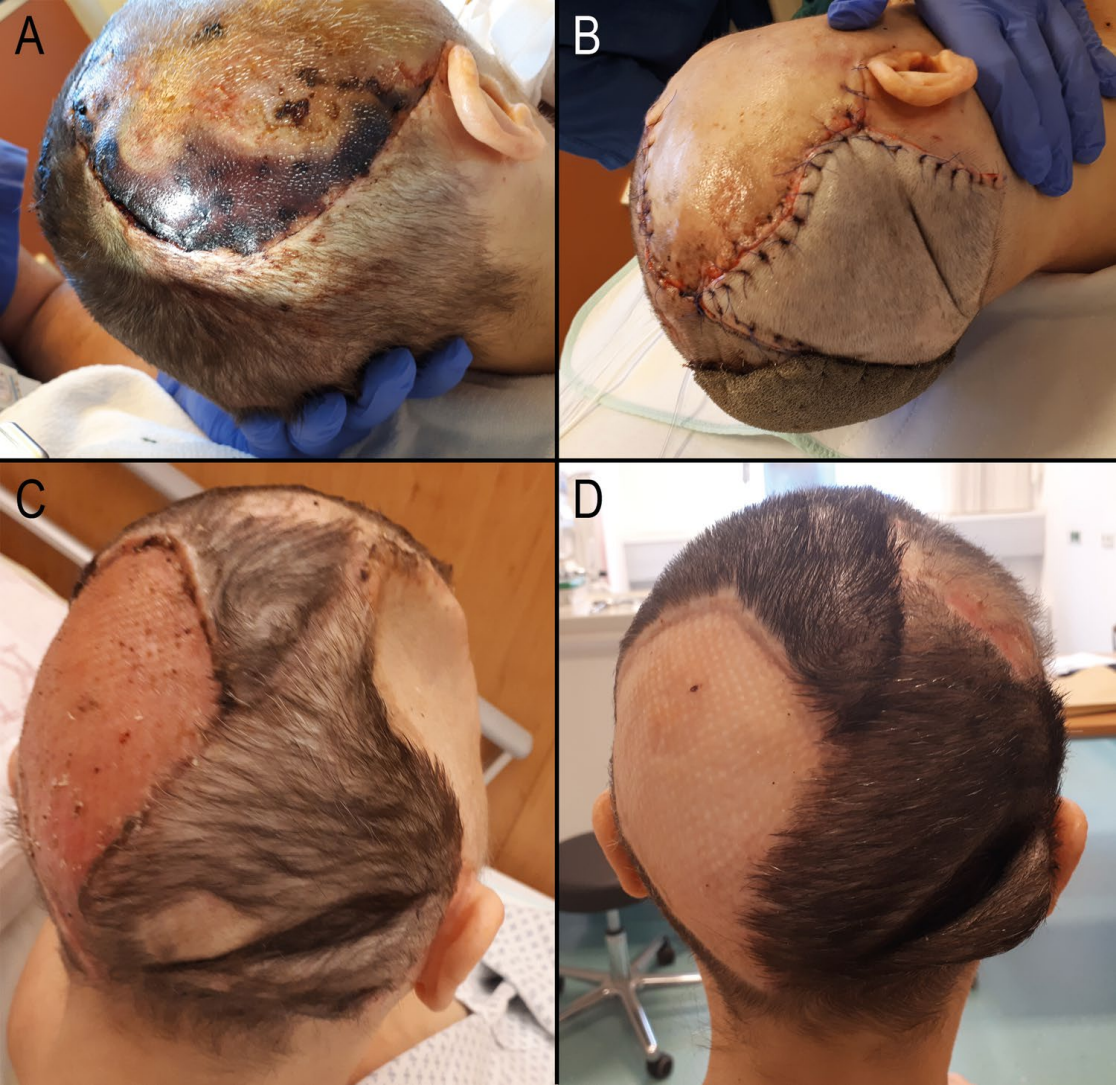
Patient 5. A 48-year-old female with severe traumatic brain injury (TBI) and a frontobasal fracture following a suicide attempt by jumping from a great height. At her most recent visit to the outpatient department, the patient was physically well, with no focal neurological deficits, but continued to exhibit psychological changes due to her preexisting depression. **A** After a question mark incision and right-sided decompressive craniectomy, a large necrotic area, approximately 38 cm², developed in the posterior part of the skin flap. Smaller necrotic areas in the frontal portion of the flap resolved spontaneously. Forty days post-TBI, a frontobasal revision and plastic surgical coverage were performed. **B** Intraoperative view after necrosectomy, frontobasal revision, and occipital rotation flap, with a split-thickness skin graft covered by a sponge. **C** Two weeks postoperatively, there was sunken skin over the bone defect and a dog-ear-like skin fold. Six months later, interdisciplinary coverage of the large bone defect was carried out. **D** Six weeks after autologous cranioplasty

**Fig. 2:**
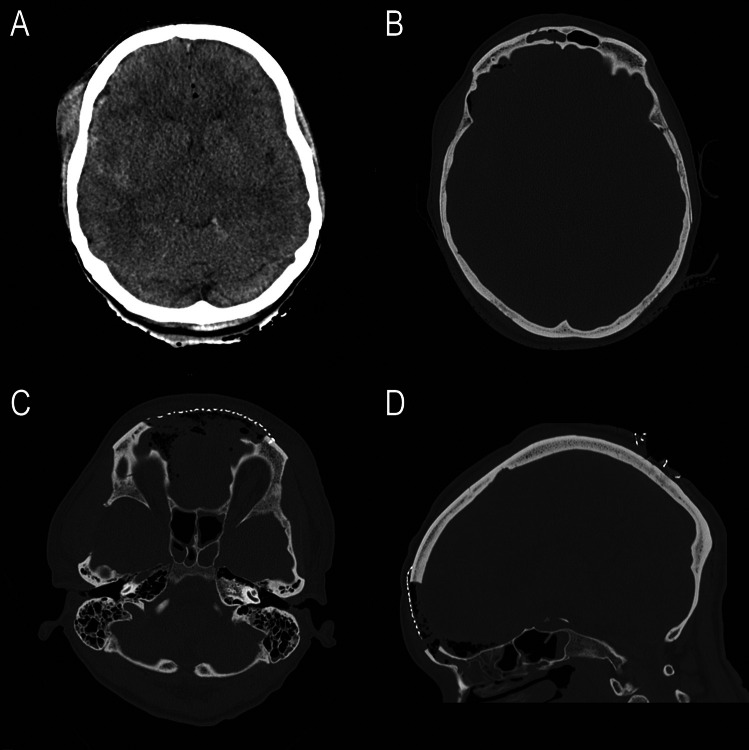
Patient 5: (**A**) initial post traumatic CT scan: brain swelling and small subdural hematoma on the right side, intracranial air (**B**) frontobasal fracture (**C**) and (**D**) postoperative CT scans, first postoperative day, frontal bone defect covered with a titanium mesh

**Fig. 3:**
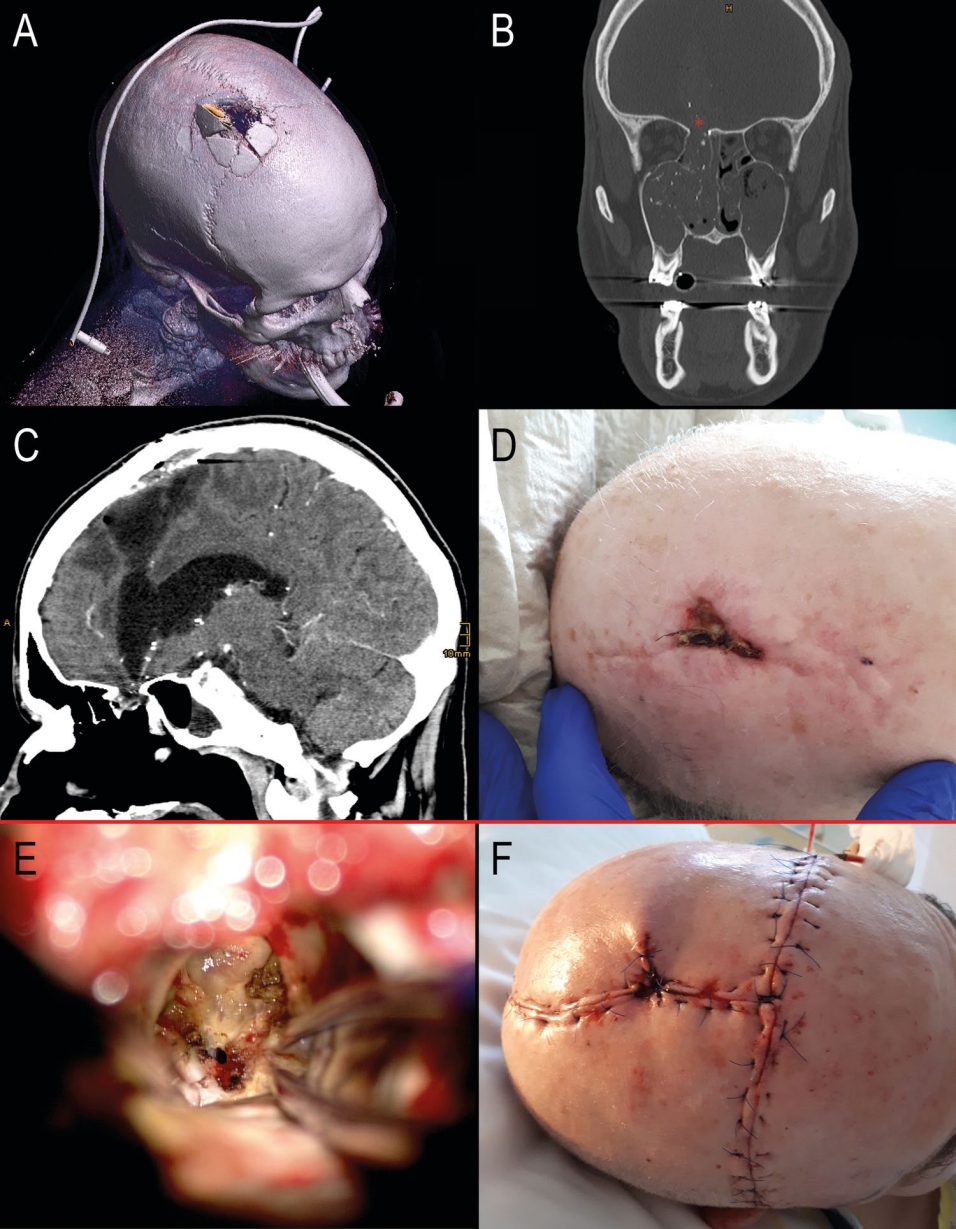
Patient 13, a 60-year-old male, sustained a self-inflicted cranial gunshot wound with the bullet entering at the base of the frontal region and exiting through the upper aspect of the frontal bone. Initially managed at an external institution, his treatment included dural closure and bone coverage with a heterologous bone graft. One month post-injury, he was transferred to our facility due to elevated infection markers, rhinoliquorrhea, and a significant brain abscess. Postoperatively, the patient showed good recovery. At the last clinical follow-up 21 months later, the patient now resides in a care facility, presenting no focal neurological deficits but continues to experience recurrent aggressive outbursts and emotional instability, due to preexisting psychiatric conditions and acquired frontal lobe damage. **A** Initial post-traumatic CT scan showing a right parasagittal bone defect at the bullet exit site. **B** Right frontobasal bone defect at the bullet entry site. **C** Intracerebral abscess formation extending from the frontobasal region to the high frontal area. **D** Preoperative view showing a 3.4 cm² frontal wound dehiscence with purulent secretion. **E** Intraoperative view of the frontobasal bone defect, accessed through the abscess from the high frontal region without necessitating a second craniotomy. Dural defects were covered with fascia lata. **F** Postoperative day 1, showing large skin incisions involving a preexisting scar

**Fig. 4:**
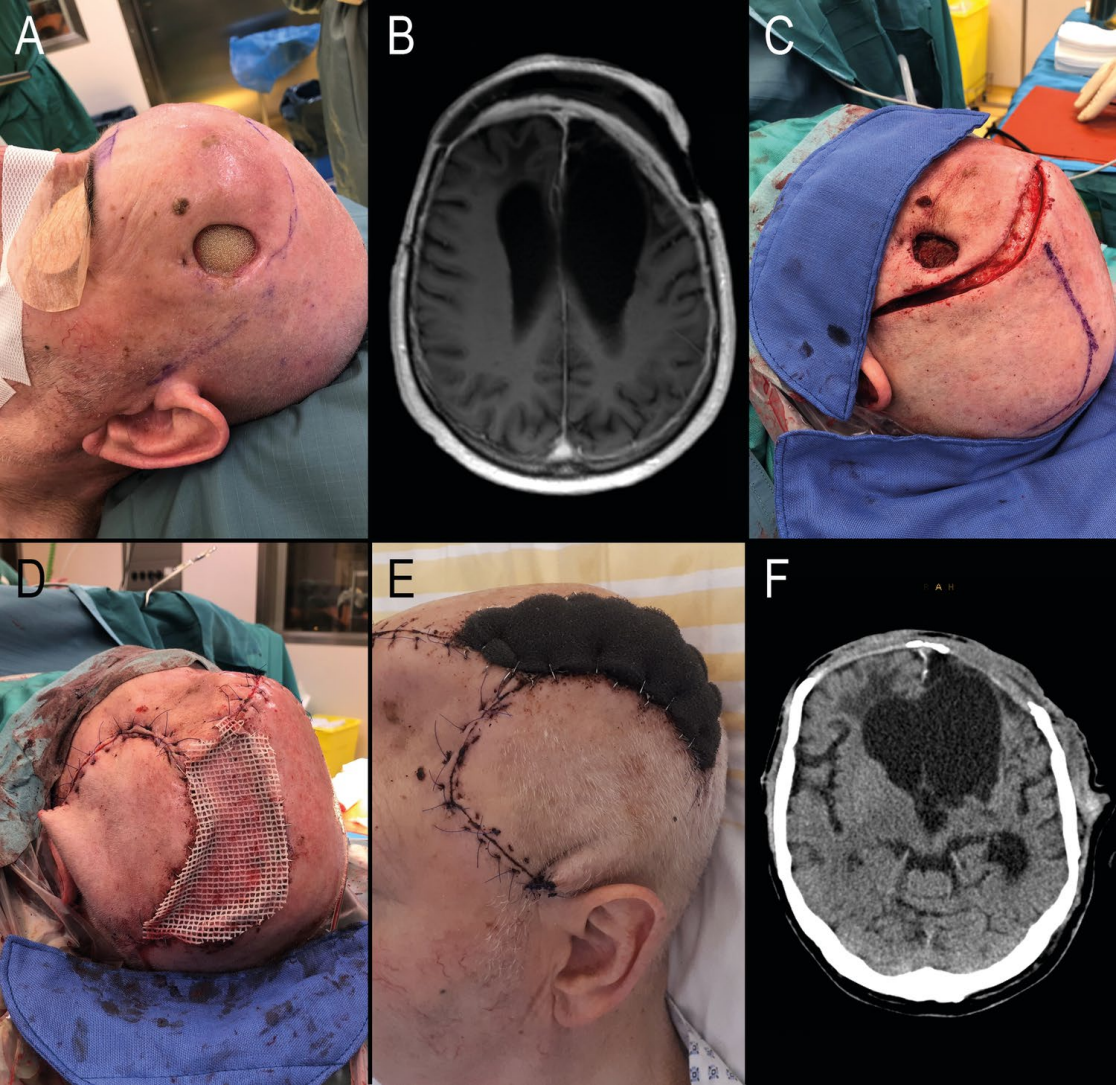
Patient 17: A 76-year-old male with a history of a large bifrontal solitary fibrous tumor (formerly known as hemangiopericytoma), classified as CNS WHO grade 2, treated with multiple surgeries for pneumocephalus and wound healing deficiencies, as well as tumor irradiation 10 years ago. The early postoperative course was uneventful. According to a follow-up telephone survey, the patient was alive 14 months after surgery but did not attend a scheduled outpatient appointment, making it impossible to evaluate his neurological status. **A** Preoperative view showing a left frontal gaping lesion of approximately 5 cm², exposing the underlying bone allograft. **B** Preoperative MRI scan revealing strong bifrontal epidural contrast enhancement, indicating infectious changes. **C** Intraoperative view after removal of the bone flap and before complete skin excision. **D** End-of-surgery view showing a left occipital rotation flap and split-thickness skin graft covered by fat gauze. **E** Early postoperative aspect with a well-vascularized skin flap and sunken skin over the bifrontal bone defect **F** Early postoperative CT scan

The following patient cases highlight the heterogeneity in causes, comorbidities, and treatments within this patient population (Figs. 1, 2, 3, 4):

### Treatment algorithm

**Fig. 5:**
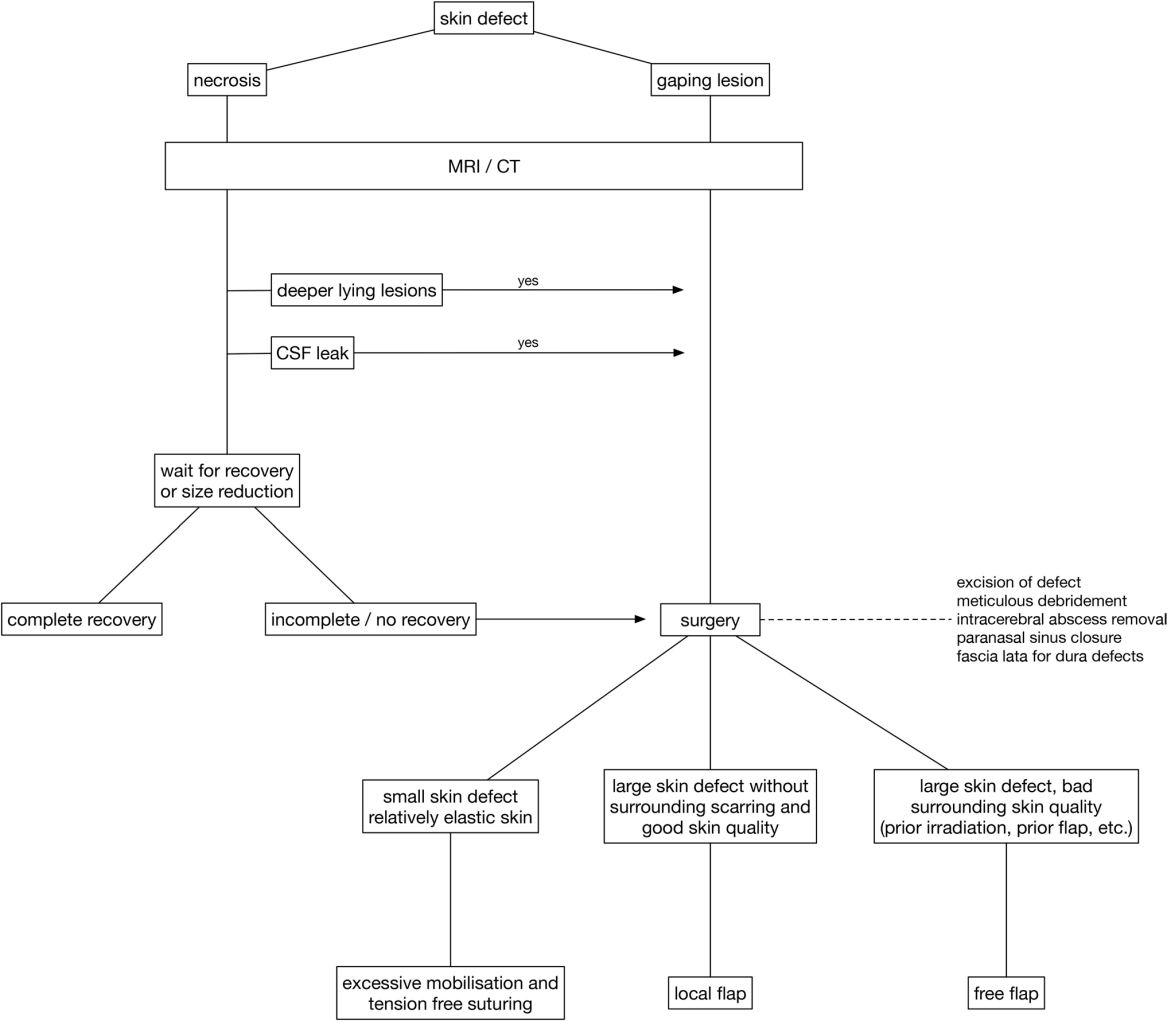
Given that it is not possible to externally determine whether areas of necrosis affect the entire thickness of the skin, we recommend a waiting period of up to 14 days in the absence of deep-seated infections or cerebrospinal fluid (CSF) leaks. This approach often results in significantly smaller necrotic areas that require excision and subsequent coverage, as full-thickness necroses are the ones that do not recover under conservative therapy. For gaping lesions we advocate an aggressive surgical approach

Based on our observations, and in accordance with principles fundamental to both neurosurgery and plastic surgery, we propose a treatment algorithm that requires further evaluation in larger cohorts (Figure 5). Since wound healing disorders requiring a multidisciplinary approach occur rarely, we invite neurosurgical colleagues to systematically analyse their cases with respect to the proposed strategy.

## Discussion

In recent years, there has been an increasing number of studies on the causes of wound healing disorders after neurosurgical interventions, especially after decompressive craniectomies and cranioplasties [4, 7, 10, 13–16, 25, 27, 29]. Skin defects may evolve as a result of ongoing chronic infection, skin thinning over implants or necrosis [4, 24, 27]. Their management can be complicated by additional pathologies such as CSF-leaks, hydrocephalus, abscesses or a reduced clinical/neurological condition. Defect sizes up to 4 cm [6] were designated as „small“ by other authors and primary wound closure was reported to be possible [9, 12]. In our series, due to chronic infection, previous radiotherapy or multiple surgeries, even minor skin defects of 0.4 cm [6] had to be closed with skin flaps in order to avoid tension on the wound margins. Whereas some other authors prefer free flaps [23, 28], our first choice were local flaps, as operating time and surgical morbidity is lower. In our experience, underlying sources of infection, especially bone flaps or allografts must be removed. Implants left in place lead to ongoing infection, repetent wound dehiscence and revision surgeries including finally also implant removal. Nevertheless, there are reports of bone - or artificial bone flaps left in place or successful immediate replacement by a new implant [3, 8, 15, 16]. Ideal timing of a subsequent cranioplasty is discussed controversely [13, 22]. According to a high implant associated failure rate in our small cohort of early performed cranioplasty, we advocate that cranioplasties following wound healing deficiencies should ideally be delayed for a minimum of six months, aiming to minimize the risk of recurrent infection.

## Conclusions

Our findings emphasize the importance of comprehensive debridement, including bone or synthetic bone flaps, tension-free wound closure, and targeted antibiotic therapy for optimal outcomes. We identify ischemic necrosis and wound dehiscence as critical factors in determining the timing of surgical intervention. Early detection of CSF leaks is crucial for evaluating the urgency and extent of surgery. A tailored, single-stage approach integrating both neurosurgical and plastic surgical techniques proves to be both effective and patient-friendly. In most cases, skin defects can be successfully addressed using local flaps. Given the complex clinical scenarios encountered in this diverse patient cohort, we advocate for a standardized, interdisciplinary approach to achieve the best results.

## Data Availability

No datasets were generated or analysed during the current study.
